# A Comprehensive Review of the Manifestations and Pathogenesis of Zika Virus in Neonates and Adults

**DOI:** 10.7759/cureus.3290

**Published:** 2018-09-12

**Authors:** Azhar Hussain, Farwa Ali, Omar B Latiwesh, Sheharyar Hussain

**Affiliations:** 1 Medicine, Xavier University School of Medicine, Oranjestad, ABW; 2 Medicine, American University of Antigua College of Medicine, New York, USA; 3 Medical Laboratory, Higher Institute of Medical Professions, Benghazi, LBY; 4 Clinical Psychology, Teachers College, Columbia University, New York, USA

**Keywords:** zika virus, vertical transmission, microcephaly, lissencephaly, ventriculomegaly and cardiovascular anomalies, congenital glaucoma

## Abstract

The Zika Virus (ZIKV) has been slowly becoming an epidemic in different parts of the world. Since its discovery in 1947, there have been numerous outbreaks affecting many different populations. Currently, there is an ongoing threat of ZIKV in Latin America and the United States of America. ZIKV is mainly spread by the *Aedes aegypti* mosquito and causes non-specific symptoms such as fever, myalgia, and generalized weakness. In addition to these symptoms, it has been noted the ZIKV is capable of causing associated conditions in adults, particularly in pregnant women as well as in newborns via vertical transmission. These manifestations include microcephaly, lissencephaly, ventriculomegaly, optic neuropathy, and congenital glaucoma, arthralgia, maculopapular rash, and cardiovascular anomalies such as atrial fibrillation. It is important to understand the reason for this specific set of associated conditions that emerge with ZIKV. This paper aims to identify the manifestations of ZIKV in adults and neonates in detail and attempts to understand the pathophysiology behind each one.

## Introduction and background

The Zika virus (ZIKV) is an arthropod-borne virus, of the genus, flavivirus. It is enveloped and spherical-shaped with an icosahedral capsid containing a non-segmented, positive-sense RNA genome. The entire structure is about 50 nm in diameter. The genome itself is made up of 10,794 bases and has two non-coding flanking regions on the 5’ and 3’ ends. There is a single open reading frame that codes for a polyprotein subsequently cleaved into capsid (C), precursor membrane (prM), envelope (E), and non-structural proteins (NS) [1]. Among these, the E protein is the most important as it is responsible for host cell binding, membrane fusion and also involved in some aspects of replication [2]. Sequencing has shown this protein to have similar homology levels to other flaviviruses [3].

ZIKV is transmitted to humans primarily through the bite of an *Aedes* mosquito, *Aedes aegypti*. In addition, unborn babies can be affected through vertical transmission in the womb. It is related to other members of the flaviviruses namely; yellow fever virus, Japanese encephalitis viruses, West Nile virus and the dengue virus [4]. ZIKV has exhibited its ability to infect in around 20% of the patients in America causing various clinical manifestations. These include acute onset of low-grade fever along with maculopapular pruritic rashes, arthralgia, and conjunctivitis [5]. ZIKV has also been related to many neurologic complications such as congenital microcephaly, Guillain-Barré Syndrome, myelitis, as well as meningoencephalitis [6] Currently, there is an outbreak of ZIKV in the United States for which the health organizations within the states have declared it, and its associated complications, as an emergency in the field of public health [4, 6].

History and epidemiology

The ZIKV was first isolated in 1947 from a serum of a sentinel rhesus macaque monkey in the Zika forest of Uganda. After a course of 60 years, the virus emerged in the Yap Island, the Federated States of Micronesia in 2007 and affected approximately 75% of the population within four months [6-7]. Between 2013 and 2014, ZIKV reached a cluster of Pacific Islands which comprised 67 islands with a total population of roughly 28,000 individuals. According to the annual epidemiological reports, approximately 11% of the total population sought medical treatment for suspected ZIKV infection [8]. The outbreak was thought to be due to the low levels of immunity and a high number of mosquitos in the area. The virus continued to spread to Latin America in 2015 and reached North America in 2016. By November 2016, the transmission of ZIKV spread over 48 countries. Territories in the Americas reported with the mosquito-borne transmission of ZIKV and a total of 171,553 confirmed cases [6-8].

## Review

**Figure 1:**
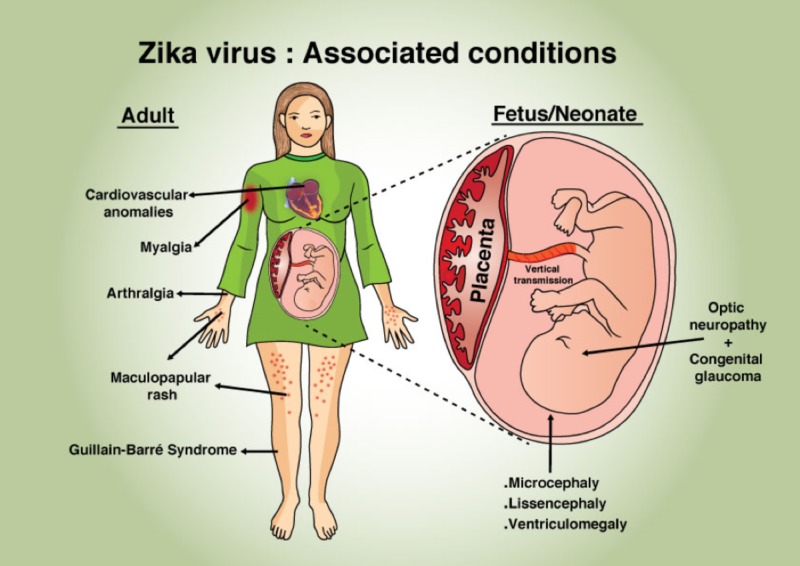
Zika associated complications in neonates and adults

ZIKV-associated symptoms, complications, and manifestations in neonates and adults are illustrated in Figure 1.

A. Life cycle of ZIKV

As mentioned earlier, ZIKV is transmitted primarily by the mosquitos of the *Aedes *genus. The *Aedes* mosquitos lay eggs in areas of high moisture which undergo metamorphosis from larvae to pupae and eventually to the adult form. This cycle takes approximately one-and-a-half to three weeks to complete. Once the female adult mosquito takes a blood meal, it can produce on average 100 to 200 eggs per batch, yielding on average five batches of eggs during her lifetime [9].

The adult female form acquires the blood to produce eggs by biting humans and animals. The virus originated in non-human primates in tropical rainforests and infected them as described in the Sylvatic cycle. The subspecies of the *Aedes *mosquitos, *Ae. africanus*,* Ae. furcifer-taylori*, *Ae. dalzieli* take blood meals from chimpanzees, monkeys, and baboons. Humans are affected when the Sylvatic cycle transitions into the Urban cycle. In this case, the subspecies of the Aedes mosquitos, *Ae. aegypti* and *Ae. albopictus*take a blood meal from humans. Lastly, it can be transmitted from human to human through sexual intercourse, blood transfusions, and in utero [2].

When the *Aedes* mosquito saliva, contaminated with ZIKV and human blood, is exchanged, the E-proteins on the surface of ZIKV are primarily involved in the attachment to the host cell membrane. The virus is internalized by endocytosis. The capsid is broken apart in the cytoplasm to release to the viral RNA. The positive sense RNA genome is translated using host ribosomes attached to the endoplasmic reticulum resulting in a polyprotein processed by proteolytic activity to structural and non-structural proteins. Once the assembly of all parts is completed, the virion is transported out of the cell using endosomal sorting complexes to the Golgi apparatus. The mature virion exits the cell by exocytosis [2, 9]. In this way, a single ZIKV can exponentially increase the quantity of the virus overwhelming the host immune defense system. 

B. Vertical transmission

The vertical mode of transmission provides passage to a disease-causing agent from mother to baby in utero or after birth. This can occur across the placenta, in the breast milk, or by direct contact during the delivery process [10]. For this to occur, the mother has to be infected during pregnancy, but before delivery [11]. ZIKV is one such pathogen that can be vertically transmitted. Although the infection itself is usually mild and self-limiting, a severe consequence of vertical in utero transmission of ZIKV is fetal/newborn microcephaly. The incidence of microcephaly in Brazil increased several times higher than in previous years with the introduction of ZIKV [12]. The Centers for Disease Control and Prevention (CDC) reported a case from January 2016 in which a baby born with microcephaly in Oahu, Hawaii, was tested positive for ZIKV infection. It was likely that the mother was infected with ZIKV in May 2015 and passed the infection to her baby [13]. This hypothesis was supported with the detection of the complete ZIKV genome and ZIKV specific IgM antibodies in the amniotic fluid of two pregnant women living in Brazil with microcephalic fetuses. This showed the virus crossed the placental barrier to infect the fetus [12]. The French Polynesia experienced one of its largest outbreaks of ZIKV since 2013. Clinical and laboratory studies done on two mothers and their newborns who had ZIKV, confirmed by reverse transcription polymerase chain reaction (RT-PCR), demonstrated further evidence of vertical transmission. RT-PCR performed on their serum samples was collected four days post-delivery. The infants were most likely to have been infected by transplacental transmission [14].

The best possibility to avoid the consequences of vertical transmission of ZIKV is to prevent the infection from occurring in pregnant women. A study conducted in Nature demonstrated how the live-attenuated ZIKV vaccine prevented viral transmission during pregnancy. This vaccine contained deletions in the 3’UTR (untranslated region) of the ZIKV genome. After a single dose of the vaccine, pregnant mice were challenged with ZIKV on embryonic day 6. An evaluation at embryonic day 13 showed markedly decreased levels of viral RNA in maternal, placental, and fetal tissues. The data collected from this study showed promising results of live-vaccines in preventing vertical transmission of ZIKV. However, the live-attenuated ZIKV vaccine is still in the phases of clinical trials [15].

C. Neonatal manifestation

Vertically transmitted ZIKV manifestations in neonates are commonly noted to be congenital microcephaly, optic neuropathy, and congenital glaucoma, ventriculomegaly, and lissencephaly. The following is an identification and discussion of each presentation of ZIKV in neonates.

Congenital Microcephaly

Congenital ZIKV can cause microcephaly and other brain abnormalities [6, 16-17]. Microcephaly is a condition in which an infant’s head circumference at birth is below the third percentile as compared to infants of the same gestational age and sex [6, 11]. Infants with microcephaly can have a wide spectrum of clinical features ranging from mild to severe; these clinical features are often lifelong because there is no cure or standard treatment option available for microcephaly. These indications include intellectual disability, seizures, movement, and balance disorders, vision or hearing problems, as well as overall developmental milestone delays in areas such as sitting, standing, or walking [8, 11, 16]. According to the CDC, microcephaly is a rare neurological disorder, affecting an estimated two infants per 10,000 live births to 12 babies per 10,000 live births each year in the United States of America [18].

The pathogenesis of postnatal microcephaly from ZIKV is not known, however; the decrease in head circumference and brain growth might be the consequence of the in-utero destruction of neuroprogenitors, neural cells, and persistent inflammatory response-associated molecules [17-18]. The inflammatory signaling pathways within the neural progenitor cells are crucial for an efficient immune response. These signaling pathways can be activated by the ZIKV, resulting in the down-regulation of the cell cycle, impaired cell-cycle progression, decrease proliferation, and neuronal apoptosis [8, 11, 17]. Pathological findings such as decreased brain volume, cerebellar hypoplasia, ventriculomegaly, subcortical and periventricular calcification, as well as cortical malformations can be seen using computed tomography (CT) scan [11, 17]. ZIKV-specific IgM can also be identified in the cerebrospinal fluid and serum of an infant with microcephaly [6]. Currently, there is no vaccine or antiviral treatment available for ZIKV and therefore, no such treatment for congenital microcephaly, which accounts for the life-threatening capability of the infection. Since treatment is not an option, prevention is the primary objective. The sole method of preventing congenital ZIKV infection is to prevent maternal infection [11]

Optic Neuropathy and Congenital Glaucoma

Optic neuropathy is defined as damage to the optic nerve, clinically manifested by partial or complete loss of vision [19]. The eyes are a very delicate organ, considered as immune-privileged organs. They are normally protected by the immune system via various factors, including blood-retinal barriers composed of tight junctions, immunosuppressive cytokines such as transforming growth factor beta (TGF-beta), inhibition of complement activation, and expression of Fas ligand (FasL) [20]. Recent studies have shown that the infection with ZIKV can cause severe ocular anomalies in infants and adults [19-20], including cellular infiltration and increased cytotoxicity and inflammation in patients [11, 20]. Most commonly, an infection with ZIKV presents as non-purulent conjunctivitis; however, serious complications including disruption of the outer macular retinal pigment epithelium, posterior uveitis, and iridocyclitis can also be a presentation [20]. The virus not only infects the optic nerve, but spreads throughout the retina infecting the ganglion, bipolar, amacrine, and Muller cells in the inner nuclear layer cells. The virus then causes thinning of the outer plexiform layer and local inflammation [6, 20], which lead to the chronic scarring of the retina [19]. The photoreceptor cells are less permissive to the virus and usually not infected; however, the infection can cause edema which can then lead to retinal folding resulting in distortion of the retina and blindness [6, 20]

Glaucoma the second leading cause of irreversible blindness in the world, affecting more than 70 million people worldwide with approximately 10% being bilaterally blind [21]. Glaucoma is characterized by a loss of retinal ganglion cells and optic disc atrophy with cupping [22-23]. Patients with glaucoma typically lose peripheral vision and if left untreated, complete vision loss occurs [23]. The pathogenesis of glaucoma is not completely understood; however, elevated intraocular pressure and vascular dysregulation are the primary contributors in the progression of glaucoma. Chronic neuropathy can also lead to glaucoma [22]. The mechanism of increased intraocular pressure can be associated with either open-angle glaucoma or closed-angle glaucoma. The primary open-angle glaucoma is usually painless and can occur with or without elevated intraocular pressure [22-23]; however, the secondary open-angle glaucoma is associated with impaired aqueous outflow due to the blockage within the drainage system of the anterior chamber angle, resulting in accumulation of aqueous fluid in the anterior chamber [21, 23]. The primary closed-angle glaucoma is due to the obstruction of the drainage pathway by the iris [21-22], resulting in the accumulation of fluid behind the iris. The patient usually complains of severe ocular pain, nausea, vomiting, and intermittent blurring of vision with halos around lights [21]. Recent studies have shown that ZIKV affects the anterior chamber of the eye during the gestation which leads to glaucoma and other associated ocular findings [24].

Ventriculomegaly

The human brain contains a set of four ventricles connected to one another. There are a set of two lateral ventricles, which are the largest in size, connected to the interventricular foramen (foramen of Monro). This foramen connects the two lateral ventricles to the third ventricle, which is subsequently connected to the fourth ventricle via the cerebral aqueduct. These are the sites of cerebrospinal fluid (CSF) production. Ventriculomegaly is the dilation of the lateral cerebral ventricles usually greater than 10mm at the level of the atria [25]. The atrium of the lateral ventricle is the portion where the body, posterior horn, and temporal horn converge. Ventriculomegaly can be an isolated event, but when it exceeds >15 mm, it is categorized as severe and is usually found to have other abnormalities associated with it such as congenital infections [26]. Among the congenital infections, ZIKV has been linked to ventriculomegaly. This is known to be a component of the Congenital Zika Syndrome (CZS). A study was conducted on two children, ages seven and eight, who had clinical presentations consistent with CZS. Their mothers lived in Cambodia, an area with known ZIKV. During their pregnancies, they experienced a fever and rash in the second trimester suggesting a ZIKV infection. These children, at birth, were born with microcephaly. In addition, infant neuroimaging demonstrated ventriculomegaly, severe cerebral atrophy, and subcortical calcifications all consistent with CZS [27].

Ventriculomegaly can result in hydrocephalus, which is an increase in pressure of the CSF within the ventricles. The increased flexibility of the skull in children allows it to be susceptible to skull enlargement secondary to increased CSF pressure. This compresses and stretches the adjacent brain tissue [28]. This results in additional associated findings as normal brain development is hindered.

Ventriculomegaly is treated after birth, usually by surgical means. The first step is to identify the etiology behind this manifestation. The next step is to take into consideration the age of the patient, rate of postoperative infection or malfunction, technical feasibility, and the great plasticity of a young brain [29]. One surgical technique is the ventriculoperitoneal (VP) shunt. This is aimed to relieve the pressure on the brain caused by excess fluid accumulation by shunting the fluid away from the cranial cavity. Under general anesthesia, the surgeon creates a small hole behind the ear by drilling into the skull. One thin catheter threads into the brain while the other catheter travels down to the chest and abdomen subcutaneously. This allows the excess CSF to drain into the abdominal cavity, which can hold a greater amount of fluid compared to the cranial cavity. The CSF is then absorbed by the body [30]. As with any surgical procedure, there are risks for which the surgeons must assess the best approach situated to each case.

Lissencephaly

The human brain consists of folds and grooves known as the sulci and gyri. The brain normally begins to fold during fetal developmental stages [31]. This requires complex signaling and expression of developmental genes for the process to occur properly. The literal definition of lissencephaly is “smooth brain”. It is a rare, gene-linked brain malformation which is characterized by the absence (agyria) or paucity (pachygyria) of these folds and grooves and microcephaly [32]. Recent outbreaks have confirmed ZIKV to be linked to congenital birth abnormalities. Neuroimaging reveals malformations of cortical development which includes lissencephaly among others [33]. As a result, the fetus/neonate will be affected depending on the degree of failure of cortical development. Some of the most common clinical manifestations include severe psychomotor retardation, developmental delay, seizures, and failure to thrive. The prognosis depends on the degree of brain malformation and severe cases can result in death in infancy or early childhood [34]. There is no cure for lissencephaly other than supportive care for basic needs. Some children can show progress in their development over time. The seizures that can be presented as a result of lissencephaly can be taken care of with anticonvulsants [32]. The other manifestations of lissencephaly are more difficult to treat with medication alone.

D. Adult manifestation

Generalized symptoms of Zika virus in adults present as conjunctivitis, mild fever, headache, skin rash, and diarrhea. However, more specifically ZIKV can manifest in adults as arthralgia, Guillain Barré Syndrome, and cardiovascular anomalies.

Arthralgia

Arthralgia is characterized by joint pain which causes injuries that affect the ligaments, bursa, or tendons surrounding the joints [35]. The pain of the condition may also cause the inflammation of the joint. ZIKV is associated with the spread of this condition. It is characterized by symptoms, such as locking of the joints, loss of the motion by the joint, and joint swelling [36]. In addition to arthralgia, patients may also experience myalgia and maculopapular rash. These symptoms are not fully understood but have a high degree of correlation with ZIKV. The rash itself is usually diffusely distributed involving the face, trunk, and extremities like palm and soles [37]. Generalized arthralgia occurs as a result of activation of nociceptors causing the release of neuromediators such as substance P and the calcitonin gene-related peptide [38]. This results in the activation of central pain pathways, as well as local sensitization of the joint receptors is the result. The specifics of the pathogenesis of arthralgia in patients with ZIKV is relatively unknown. Certain studies associate accreditation of arthralgia to older age, a comorbid illness, or the ongoing viremia leading to the immune responses in ZIKV infected patients [39].

Guillain-Barré Syndrome (GBS)

In the recent researches conducted, it has been proven that the relationship between the ZIKV and the nervous system, known as the Guillain-Barré Syndrome, has risen in different countries especially in America. GBS is a demyelinating condition in which the body's immune system attacks the peripheral nervous system [40-41]. This occurs typically a few days after the body has been exposed to the virus, parasite or even some bacteria. If this condition is not treated in a timely manner, a patient can die from complications, such as blood infections, lung clots, cardiac arrest and finally the paralysis of the muscles that generally monitors the breathing [42]. Guillain Barré Syndrome is essentially an autoimmune condition; hence the pathophysiology of the condition reflects an immune response. As mentioned previously, GBS is caused by a cell-mediated immune response on peripheral nerve myelin proteins [43]. The most accepted theory behind the mechanism of GBS is that the infectious pathogen is composed of amino acids which mimic the peripheral nerve myelin protein. When the immune system responds to the pathogenic amino acids, it is not able to distinguish between the pathogen and the normal peripheral nerve myelin protein. Therefore, this autoimmune disease results in the self-destruction of peripheral nerve myelin, most likely the ganglioside GM1b [44]. The autoimmune attack consequently results in an influx of immune mediated-agents and macrophages resulting in inflammation and destruction of the axon, leaving it unable to support nerve conduction. This immune response is triggered by a variety of pathogens. Recent studies have noted a positive correlation between adult patients infected with ZIKV and the chances of it manifesting as GBS. A study was done with 39 patients that had a confirmed GBS diagnosis. After comparing cases-control, the increased risk factor for GBS in ZIKV patients was an acute illness within the previous two months [41]. A high degree of prevention of ZIKV should be observed in epidemic places after an acute illness with bacteria or virus [6].

Cardiovascular Anomalies

Atrial fibrillation, a cardiovascular condition, involves a rapid heartbeat leading to an increased risk of contracting stroke, heart failure, and other complications [45]. The pathophysiology involves the two upper chambers of the heart beating irregularly without synchronization, leading to loss of coordination between the upper and lower chambers. ZIKV aids in the incidence and progression of this condition. Some of the symptoms of these conditions include shortness of breath, general body weakness, and palpitations [46]. This associated complication with ZIKV may be episodic resolving with the resolution of the disease or it may be chronic. If chronic, long-term treatment is warranted. It is typically fatal and requires medication. Its treatment can be limited to medication only or even include other interventions that may alter the heart’s electrical system [47].

The association with atrial fibrillation and other cardiovascular (CVS) complications was made in a study comprised of adults with no prior history of CVS disease in Caracas, Venezuela, an epicenter of ZIKV outbreaks. The study resulted in all but one patient developing a dangerous heart rhythm condition and two-thirds of the subjects showed evidence of heart failure [48].

E. Current treatment options

ZIKV has been making headlines in different countries due to its causal relationship to the microcephaly, severe brain defects along with diseases that severely affects the nervous system in unborn babies especially if the pregnant mothers who contract the virus [49]. This is a major concern for the CDC. The first step to preventing any infection is to prevent contact. The mosquitos cause and spread disease when they come in contact with host blood supply. This can be prevented with the installation of different mosquito control vectors to curb the spread of the diseases related to the infection [6, 8, 48]. Pregnant women and individuals at high risk should receive proper medical care to control this infection. The best option is to seek medical assistance for the clinical care [50].

Symptomatic treatments of ZIKV associated conditions are also available, such as the VP shunt in microcephaly, drugs to treat heart rhythm problems, pain medication for arthralgia, glaucoma management, and other palliative treatment options. Conditions like microcephaly or lissencephaly are much more difficult to treat as they are congenital and require a more invasive approach.

## Conclusions

ZIKV is spread by the *Aedes* mosquito and its associated conditions can be fatal. Once the virus has infected an individual, it has no specific treatments, and only requires proper medical care. The diseases associated with the virus mainly infects the unborn fetus due to their weak immune system. This occurs after the virus carrying *Aedes *mosquito bites a pregnant woman. A majority of the people who are infected by this virus do not develop symptoms. If symptoms develop, they are initially generalized, presenting as fever, rash, and headaches among others. The virus has the ability to create various complications during pregnancy, but also in non-pregnant adults.

ZIKV needs to be studied further to determine the exact mechanism through which these conditions arise. The virus is very likely to migrate beyond the Americas, and this research will attempt to provide the pathophysiology to eventually aid in the prevention of these associated conditions.

## References

[1] Faye O, Freire CCM, Iamarino A (2018). Molecular evolution of Zika virus during its emergence in the 20th century. PLoS Negl Trop Dis.

[2] Kraemer MU, Sinka ME, Duda KA (2018). The global distribution of the arbovirus vectors Aedes aegypti and Ae. albopictus. eLife.

[3] Kostyuchenko VA, Lim E, Zhang S (2016). Structure of the thermally stable Zika virus. Nature.

[4] Mittal R, Nguyen D, Debs LH (2017). Zika virus: an emerging global health threat. Front Cell Infect Microbiol.

[5] Braga JU, Bressan C, Dalvi APR (2018). Accuracy of Zika virus disease case definition during simultaneous Dengue and Chikungunya epidemics. PLoS One.

[6] Song B, Yun S, Woolley M, Lee Lee, Y Y (2017). Zika virus: History, epidemiology, transmission, and clinical presentation. J Neuroimmunol.

[7] (2018). The history of Zika virus. WHO.

[8] Musso D, Roche C, Robin E, Nhan T, Teissier A, Cao-Lormeau VM (2015). Potential sexual transmission of Zika virus. J Emerg Infect Dis.

[9] Wang B, Tan XF, Thurmond S (2018). The structure of Zika virus NS5 reveals a conserved domain conformation. Nat Commun.

[10] Leeper C, Lutzkanin A 3rd (2018). Infections during pregnancy. Prim Care.

[11] Chen HL, Tang RB (2016). Why Zika virus infection has become a public health concern?. J Chin Med Assoc.

[12] Bordi L, Avsic-Zupanc T, Lalle E, Vairo F, Capobianchi MR, da Costa Vasconcelos PF (2016). Emerging Zika virus infection: a rapidly evolving situation. Adv Exp Med Biol.

[13] Rabaan AA, Bazzi AM, Al-Ahmed SH, Al-Ghaith MH, Al-Tawfiq JA (2017). Overview of Zika infection, epidemiology, transmission and control measures. J Infect Public Health.

[14] Besnard M, Lastere S, Teissier A, Cao-Lormeau VM, Musso D (2018). Evidence of perinatal transmission of Zika virus, French Polynesia, December 2013 and February 2014. Euro Surveill.

[15] Shan C, Muruato AE, Jagger BW (2018). A single-dose live-attenuated vaccine prevents Zika virus pregnancy transmission and testis damage. Nat Commun.

[16] Cauchemez S, Besnard M, Bompard P (2016). Association between Zika virus and microcephaly in French Polynesia, 2013-15: a retrospective study. Lancet.

[17] van der Linden V, Pessoa A, Dobyns W (2018). Description of 13 infants born during October 2015-January 2016 with congenital Zika virus infection without microcephaly at birth — Brazil. MMWR Morb Mortal Wkly Rep.

[18] (2018). Facts about microcephaly. Centers for Disease Control and Prevention. Cen Dis Cont Prev.

[19] De Moraes CG, Pettito M, Yepez JB (2018). Optic neuropathy and congenital glaucoma associated with probable Zika virus infection in Venezuelan patients. J Med Microbiol Case Rep.

[20] Manangeeswaran M, Kielczewski JL, Sen HN (2018). ZIKA virus infection causes persistent chorioretinal lesions. Emerg Microbes Infect.

[21] Weinreb RN, Aung T, Medeiros FA (2014). The pathophysiology and treatment of glaucoma: a review. JAMA.

[22] Agarwal R, Gupta SK, Agarwal P, Saxena R, Agrawal SS (2009). Current concepts in the pathophysiology of glaucoma. Indian J Ophthalmol.

[23] Kwon YH, Fingert JH, Kuehn MH, Alward WLM (2009). Primary open-zngle glaucoma. N Eng J Med.

[24] Greenwood M (2018). Zika and glaucoma linked for first time in new study. YaleNews. YaleNews.

[25] Dantonio F, Papageorghiou AT (2018). Ventriculomegaly. Obstetric Imaging: Fetal Diagnosis and Care (Second Edition).

[26] Norton ME (2018). Fetal cerebral ventriculomegaly. UpToDate. UpToDate.

[27] Chu V, Petersen LR, Moore CA (2018). Possible congenital Zika syndrome in older children due to earlier circulation of Zika virus. Am J Med Genet A.

[28] McAllister JP 2nd (2012). Pathophysiology of congenital and neonatal hydrocephalus. Semin Fetal Neonatal Med.

[29] Wang K, Lee JY, Kim S, Phi JH, Cho B (2011). Fetal ventriculomegaly: postnatal management. Childs Nerv Syst.

[30] Roth E (2018). Ventriculoperitoneal shunt. Healthline. healthline.

[31] Nall R (2018). Lissencephaly. Healthline. healthline.

[32] (2018). Lissencephaly information page. National Institute of Neurological Disorders and Stroke. Nat Inst Neuro Dis Str.

[33] Mehrjardi MZ, Keshavarz E, Poretti A, Hazin AN (2018). Neuroimaging findings of Zika virus infection: a review article. Jpn J Radiol.

[34] Ghai S, Fong KW, Toi A, Chitayat D, Pantazi S, Blaser S (2006). Prenatal US and MR imaging findings of lissencephaly: review of fetal cerebral sulcal development. J Cont Med Edu Radio.

[35] Irwin ML, Cartmel B, Gross CP (2015). Randomized exercise trial of aromatase inhibitor-induced arthralgia in breast cancer survivors. J Clin Oncol.

[36] Tamaki K, Takaesu M, Nagamine S (2018). Abstract P6-11-01: Final results of the randomized trial of exercise intervention vs. usual care for breast cancer patients with aromatase inhibitor to prevent and improve the aromatase inhibitor induced arthralgia. Cancer Res.

[37] Navalkele BD, Levine MT, Chandrasekar PH (2018). Zika virus clinical presentation. Medscape. Medscape.

[38] Perrot S, Guilbaud G (1996). Pathophysiology of joint pain. Rev Rhum.

[39] Edupuganti S, Natrajan MS, Rouphael N (2018). Biphasic Zika Illness with rash and joint pain. Open Forum Infect Dis.

[40] Cao-Lormeau VM, Blake A, Mons S (2016). Guillain-Barré Syndrome outbreak associated with Zika virus infection in French Polynesia: a case-control study. Lancet.

[41] Leis AA, Stokic DS (2018). Zika virus and Guillain-Barre Syndrome: is there sufficient evidence for causality?. Front Neurol.

[42] Parikh V, Tucci V, Galwankar S (2012). Infections of the nervous system. Int J Crit Illn Inj Sci.

[43] Kuwabara S (2004). Guillain-Barré syndrome: epidemiology, pathophysiology and management. Drugs.

[44] Heikema AP, Koning RI, dos Santos Rico SD (2013). Enhanced, sialoadhesin-dependent uptake of Guillain-Barré Syndrome-associated Campylobacter jejuni strains by human macrophages. Infect Immun.

[45] Calkins H, Hendricks G, Cappato R (2017). 2017 HRS/EHRA/ECAS/APHRS/SOLAECE expert consensus statement on catheter and surgical ablation of atrial fibrillation. Heart Rhythm.

[46] January CT, Wann LS, Alpert JS (2014). 2014 AHA/ACC/HRS guideline for the management of patients with atrial fibrillation: executive summary: a report of the American College of Cardiology/American Heart Association Task Force on practice guidelines and the Heart Rhythm Society. Circulation.

[47] Verma A, Jiang CY, Betts TR (2015). Approaches to catheter ablation for persistent atrial fibrillation. N Engl J Med.

[48] Santye L (2018). Zika virus may be linked to cardiovascular complications. Contemporary Clinic. Cont Clin.

[49] Mumtaz N, van Kampen JJ, Reusken CB, Boucher CA, Koopmans MPG (2016). Zika virus: where is the treatment?. Curr Treat Options Infect Dis.

[50] Heymann DL, Hodgson A, Freedman DO (2016). Zika virus and microcephaly: why is this situation a PHEIC?. Lancet.

